# Characterization of preantral follicle clustering and neighborhood patterns in the equine ovary

**DOI:** 10.1371/journal.pone.0275396

**Published:** 2022-10-04

**Authors:** Kendall A. Hyde, Francisco L. N. Aguiar, Paula B. Alvarenga, Amanda L. Rezende, Benner G. Alves, Kele A. Alves, Gustavo D. A. Gastal, Melba O. Gastal, Eduardo L. Gastal

**Affiliations:** 1 Animal Science, School of Agricultural Sciences, Southern Illinois University, Carbondale, Illinois, United States of America; 2 Department of Veterinary Medicine, Sousa Campus, Federal Institute of Education, Science and Technology of Paraíba, Sousa, Paraíba, Brazil; 3 Instituto Nacional de Investigación Agropecuaria, Estación Experimental INIA La Estanzuela, Colonia, Uruguay; China Agricultural University, CHINA

## Abstract

Understanding the transition from quiescent primordial follicles to activated primary follicles is vital for characterizing ovarian folliculogenesis and improving assisted reproductive techniques. To date, no study has investigated preantral follicle crowding in the ovaries of livestock or characterized these crowds according to follicular morphology and ovarian location (portions and regions) in any species. Therefore, the present study aimed to assess the crowding (clustering and neighborhood) patterns of preantral follicles in the equine ovary according to mare age, follicular morphology and developmental stage, and spatial location in the ovary. Ovaries from mares (n = 8) were collected at an abattoir and processed histologically for evaluation of follicular clustering using the Morisita Index and follicular neighborhoods in ovarian sections. Young mares were found to have a large number of preantral follicles with neighbors (n = 2,626), while old mares had a small number (n = 305). Moreover, young mares had a higher number of neighbors per follicle (2.6 ± 0.0) than old mares (1.2 ± 0.1). Follicle clustering was shown to be present in all areas of the ovary, with young mares having more clustering overall than old mares and a tendency for higher clustering in the ventral region when ages were combined. Furthermore, follicles with neighbors were more likely to be morphologically normal (76.5 ± 6.5%) than abnormal (23.5 ± 6.5%). Additionally, morphologically normal activated follicles had increased odds of having neighbors than normal resting follicles, and these normal activated follicles had more neighbors (2.6 ± 0.1) than normal resting follicles (2.3 ± 0.1 neighbors). In the present study, it was demonstrated that preantral follicles do crowd in the mare ovary and that clustering/neighborhood patterns are dynamic and differ depending on mare age, follicular morphology, and follicular developmental stage.

## Introduction

The oocyte reserve in the ovaries of mammals is represented by a large but finite pool of quiescent, primordial follicles. Over time, these primordial follicles may be activated to grow, with the goal of producing a fertilizable oocyte [1], or become atretic and die [2]. Via either path (ovulation or atresia), the oocyte reserve will eventually be depleted until ovarian senescence occurs. Due to this fact, it is imperative to improve knowledge surrounding follicular activation with the aim to recover the preantral follicle population before depletion, potentially extending female reproductive lifespan via assisted reproductive techniques (ARTs). In this regard, the mare, despite possessing unique ovarian anatomy, has become a powerful animal model due to its capability to provide insight into ovarian function and physiology for women and other livestock species (for review, see [3–6]).

One of the key processes in folliculogenesis is the activation of primordial follicles to develop into primary follicles. During this process, primordial follicles in the quiescent pool, organized in crowded clusters, leave the pool and start to develop into later stages of preantral follicles (i.e., primary, secondary). This developmental activation is modulated by suppressive and maintenance factors (i.e., transforming growth factor, anti-Müllerian hormone, kit ligand, growth differentiation factor) produced by nearby follicles and surrounding cells (for review, see [7–9]). In this context, the paracrine communication between preantral follicular cells may have a putative association with the potential location of these follicles in the ovary.

With respect to location, it is well-documented that the distribution of preantral follicle population in the ovary is heterogeneous in many species (mare: [10]; woman: [11]; cow: [12]; mouse: [13]; deer: [14]; ewe: [15]; doe: [16]). This heterogeneity may partially be explained by the working hypothesis of ovarian plasticity, which proposes that preantral follicles migrate throughout the ovarian cortex during follicular development [17]. In this regard, in the mare, during follicular developmental activation, it has been suggested that preantral follicles migrate toward the geometric center of the ovary [18], while migration may cease once a follicle becomes morphologically abnormal (a potential sign of atresia; [19]). Furthermore, as reported by Gaytan *et al*. [20], primordial follicles tend to cluster with nearby follicles in the ovaries of mice and women; meanwhile, these clustering patterns are less often seen for growing follicles. This finding is suggestive of the concept that paracrine signals in the ovary play a large role in follicular activation and the development of primordial follicles [20]. The potential paracrine activity within follicle clusters and the hypothesis of ovarian plasticity may work together to explain the wide range of follicular heterogeneity observed in the mare, as well as the follicular decrease that occurs during mare aging [19, 21, 22].

Based upon the available information concerning the equine ovary as well as that of other species, the hypothesis of the present study is that equine preantral follicles are organized in clusters within the ovary and the patterns of these clusters depend upon mare age and ovarian spatial location. Therefore, the current study aimed to assess the crowding/clustering patterns of preantral follicles in the mare ovary according to (i) mare age, (ii) developmental stage of the follicle, (iii) follicular morphology, (iv), and spatial location in the ovary. A novel study that characterizes the clustering patterns of equine preantral follicles that combines analysis of follicle morphology and the influence of mare age warrants scientific investigation. Perhaps by elucidating details surrounding follicular morphology, clustering, and spatial location, improvements to *in vitro* processes such as follicular culture or even creation of an artificial ovary may be made. If the ideal scenario for maintenance of normal primordial follicles long-term or for controlled activation of these follicles can be achieved, the contributions to ARTs and extending female reproductive lifespan would be substantial.

## Materials and methods

### Ovaries

**Fig 1:**
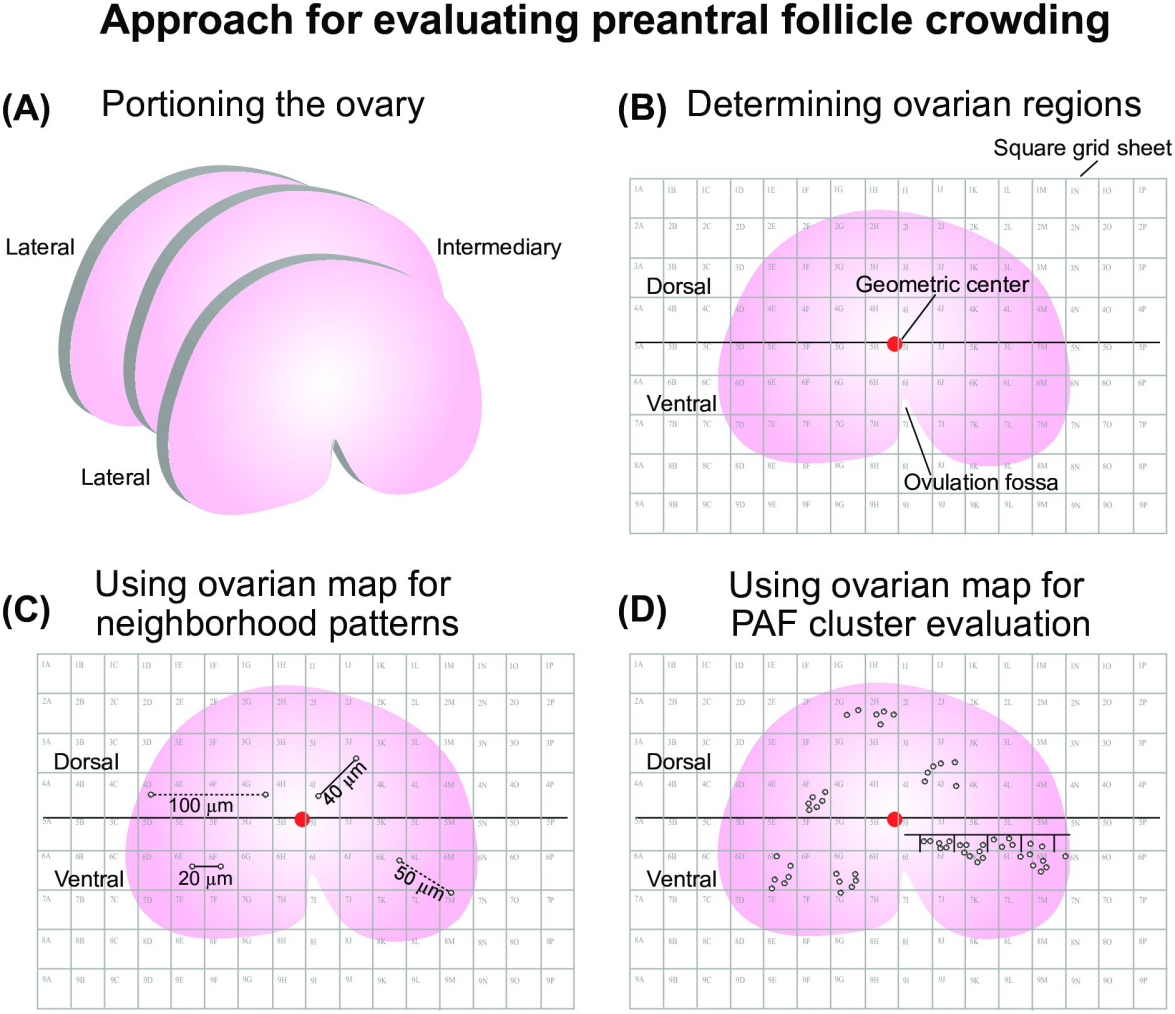
Illustration of procedures performed to assess the neighborhood and clustering patterns of equine preantral follicles. (A) Ovaries were divided into three portions (lateral, n = 2; intermediary, n = 1) and then histologically processed. Once sections of the ovaries were mounted onto microscope slides, (B) a square grid sheet (area of each square = 0.0625 cm^2^) with columns and rows labelled with numbers and letters, respectively, was placed onto each slide. The geometric center was then determined, and a longitudinal line was drawn through the center to define the dorsal (all areas above the longitudinal line) and ventral (all areas below the longitudinal line) regions. From this point on, the slide is referred to as an “ovarian map.” Ovarian maps were used to evaluate (C) neighborhood patterns, where preantral follicles within 40 μm of each other (indicated by solid lines) were considered to be neighbors, while follicles further than 40 μm were not considered to be neighbors (indicated by dashed lines), and (D) clustering, which was calculated considering the number of follicles in five consecutive T-shaped plots (not to scale) using the formula for Morisita’s index of clustering.

Ovaries of mixed-breed, light horse mares were collected during the middle of the reproductive season at an equine slaughterhouse (Frigorifico Foresta Ltda.) in Brazil (30° 20’ 38” S, 54° 20’ 31” W). The ovaries (Fig 1) were then separated into two age groups (young: 4–9 years and old: ≥20 years; *n* = 4 pairs of ovaries for each group) using dental characteristics. Immediately upon collection, the ovaries were sectioned into three longitudinal portions (2 lateral portions, 1 intermediary portion; Fig 1A; [18]). The sections were fixed in 4% paraformaldehyde for 24 hours, then stored in 70% alcohol until histological processing. Reproductive status of the mares (anestrus or cycling) was unknown, and only ovaries without visible preovulatory follicles and/or corpora lutea were selected.

### Histological processing

The fixed ovarian portions were dehydrated, embedded in paraffin wax, and completely cut into 7 μm serial sections [23]. To prevent double counting of follicles, every 5th section was then mounted on large (127 x 102 mm) microscope slides for visualization of the whole ovary. Mounted sections with intact ovulation fossa and clear borders without lacerations were then chosen to ensure good tissue quality for analysis. Afterward, the slides were stained with periodic acid-Schiff (PAS) and counterstained with hematoxylin to be used for microscopic evaluation.

### Production of ovarian maps

To determine the spatial distribution of the follicles within ovarian portions and regions (dorsal and ventral; [18]), a square grid sheet (area of each square = 0.0625 cm^2^) with columns labeled with letters and rows labeled by numbers was placed over the slides (Fig 1B). The square grid sheet was aligned to the upper-left corner of each slide with the ovulation fossa facing the bottom of the square grid sheet. Images of all mounted histological sections were captured and digitized in a photo-editing program (Adobe Photoshop CS4; San Jose, USA). Next, a longitudinal line was drawn through the mathematically determined geometric center [19] using a marking tool in the editing program (Adobe Photoshop CS4), dividing the ovary into dorsal (above the longitudinal line) and ventral (below the longitudinal line, containing the ovulation fossa) regions. Once follicles were identified via light microscopy (Nikon Eclipse E200; Tokyo, Japan), the 2-dimensional location of each follicle was noted using the *x*- and *y*-coordinates (rows and columns) provided by the square grid sheet, with the slide now being referred to as an ovarian map.

### Preantral follicle morphology and classification

The morphometric patterns for preantral follicle classification were performed as previously described [21, 23]. Follicles were classified as normal (intact, non-pyknotic oocyte nucleus and surrounded by well-organized granulosa cells) or abnormal (retracted ooplasm, disorganized granulosa cells, and a pyknotic nucleus). Furthermore, follicles were classified according to developmental stage as primordial (single layer of flattened granulosa cells), transitional (single layer of both flattened and cuboidal granulosa cells), primary (single layer of cuboidal granulosa cells), or secondary (two or more layers of cuboidal granulosa cells). Thus, both morphologically normal and abnormal follicles were further classified as resting (primordial) or activated (transitional, primary, and secondary).

### Neighborhood pattern identification and assessment

**Fig 2:**
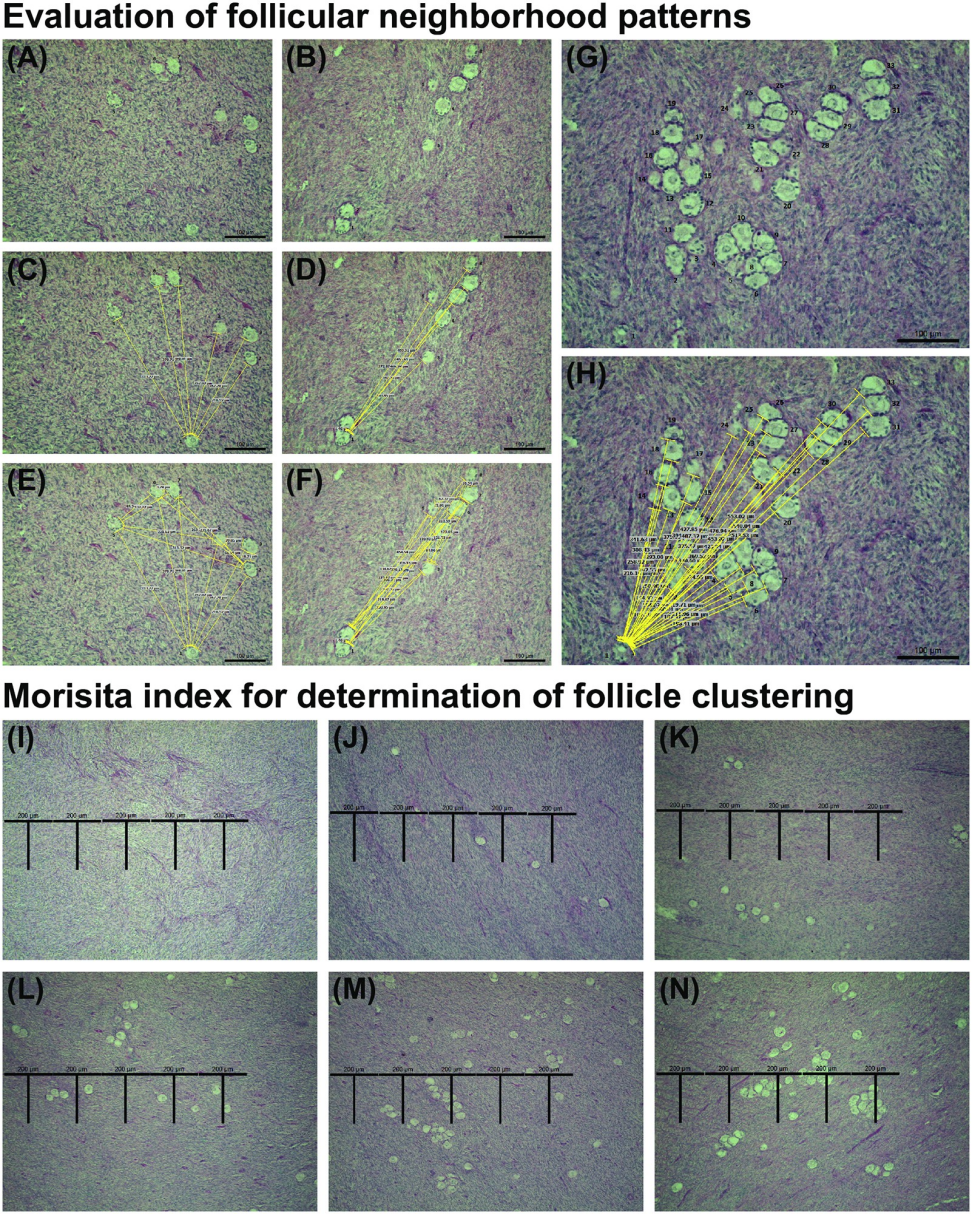
Images of experimental procedures performed to assess the preantral follicle neighborhood and clustering patterns. (A–H) Representative images used for determination of follicular neighborhood patterns are shown. For neighborhood pattern assessment, random fields were imaged, and (A, B, G) follicles within each image were numbered, and classified according to morphology and developmental stage. Next, (C, D, H) the distances from the first follicle (labelled with the number “1”) were measured, and subsequently, (E, F) the distances between all follicles within the field were measured. Scale bars = 100 μm, magnification = 20×. (I–N) Representative images of randomly generated fields depicting progressive lower to higher clustering patterns are also shown. To assess preantral follicle clustering, the fields were overlaid with 5 consecutive 200 x 200 μm “T” plots, the number of follicles in each plot were counted, and the clustering index was calculated. Scale bars = 200 μm, magnification = 10×.

For neighborhood analysis, images were captured from 20 random fields in each histological section/ovarian map (10 images in the dorsal and 10 images in the ventral regions). The method for image capturing consisted of locating the longitudinal line dividing the histological section into dorsal and ventral regions. The first square on the left side of the ovarian map (above the line: dorsal; below the line: ventral) that had ovarian tissue was used as a starting point. Next, the ovarian map was searched, going square-by-square, to find the first square with preantral follicles, which was then captured as the first image (Fig 1C). From this point onward, the knob of the microscope stage was completely turned three times, and the location that resulted from this action was then captured as the next image. This process occurred 10 times in each ovarian region per histological section to obtain 20 images for each slide. These images (Fig 2) were obtained using Leica Application Suite software (Leica Imaging Software, Wetzlar, Germany) coupled to a properly calibrated light microscope (Nikon) at 20× magnification. Subsequently, follicles within each image were numbered (Fig 2A, 2B and 2G) and analyzed using ImageJ software (ImageJ, version 1.45; National Institute of Health, USA) calibrated (150 pixels = 100 μm) to measure the distances between follicles. Initially, the lowest and left-most follicle in each image was chosen as the “anchoring” follicle, and the distances from the anchoring follicle to all other follicles in the image were measured and recorded (Fig 2C, 2D and 2H). Subsequently, the distances between all follicles in the microscopic field were measured (Fig 2E and 2F). This process was repeated for every follicle in the image until all distances between follicles were measured and recorded. Follicles were considered neighbors when they were ≤40 μm apart from each other [20]. A 40 μm radius between follicles is considered to be a conservative distance over which diffusible signals can travel in the ovary [20, 24].

### Evaluation of preantral follicle clustering

To assess if the clustering pattern of preantral follicles is influenced by age and the ovarian region and portion in which they are found, the Morisita index of clustering methodology was applied [20, 25]. This index consists of a dispersion measure, capable of evaluating individual variations in a population (ecological studies: [25]; brain tissue: [26]; ovarian tissue: [20]; fascial tissue: [27]). Using light microscopy (Nikon), 5 consecutive, T-shaped plots (200 x 200 μm in a 10× objective) in 10 random fields of each ovarian region (dorsal and ventral) per histological section were image-captured (Leica Imaging Software; Fig 2I–2N), and any follicles within the plots were counted (50 plots per region, 100 plots in total per section; Fig 1D). The images for this analysis were captured according to the same methodology used in the neighborhood analysis, in which the first image was captured in the first square from the left, above and below the mid-longitudinal line. The other images were obtained after three complete turns of the knob controlling the microscope stage location.

The Morisita index (I_δ_) was obtained using the following equation: I_δ_ = q {[Σ(xi^2^)–Σxi]/[(Σxi)^2^ –Σxi]}, where q = the number of plots evaluated per region per section (5 plots per image, 10 images per region to generate 50 plots for evaluation per region per section in this work); xi = the number of follicles per plot; Σxi = the sum of the number of follicles in each plot; Σ(xi^2^) = the sum of squares of the number of follicles in each plot; (Σxi)^2^ = the sum of the number of follicles in each plot, squared.

### Statistical analyses

All statistical analyses were performed using R statistical software, version 4.1.3 (R Foundation for Statistical Computing, Vienna, Austria). Percentages were transformed using arc sin square root, while any non-percentage data determined to be non-normally distributed (Kolmogorov-Smirnov test) were transformed using base 10 logarithm (log_10_). One-ANOVA followed by *post hoc* Fisher’s Least Significant Difference tests were used for multiple comparisons of means, while any data found to be non-normal after transformation were analyzed using non-parametric tests (Kruskal-Wallis and *post hoc* Dunn’s tests). Confidence Intervals (95%) were determined for the relative probabilities of activation and odds ratios to compare the association between presence of follicle neighbors and follicular stage. Significances for Morisita indices were determined using the equation Z = (Iδ− 1)/(2/qm^2^)^0.5^, where m corresponds to the mean number of follicles. Clustering was determined to be significant for Z ≥ 1.96 [28], meaning that there was a clustering pattern rather than a random pattern of spatial distribution. Data were determined to be significant when *P* < 0.05 and tended to be significant when 0.05 < *P* < 0.1.

## Results

### Preantral follicle morphology and classification

A total of 206 ovarian histological sections/ovarian maps were evaluated (25.8 ± 1.1 sections per mare), with a total of 3,476 preantral follicles recorded. From these follicles, 2,014 primordial, 1,460 transitional, and two primary follicles were identified. The numbers and percentages of observed preantral follicles according to mare ages, ovarian locations (portions and regions), and follicular developmental stages are shown (Table 1). A wide range and large number of preantral follicles were observed in the young mares (e.g., 0–453 and 3,024), while a narrow range and small number were observed in the old mares (e.g., 0–191 and 452).

**Table 1:** Descriptive percentages and numbers of observed follicles according to mare ages, ovarian locations (portions and regions), and follicular developmental stages, and mean percentages (± SEM) and numbers (± SEM) of observed follicles according to ovarian locations and follicular developmental stages.

Age group		Ovarian location (portion and region)									
		Lateral dorsal		Lateral ventral		Intermediary dorsal		Intermediary ventral		Overall	
		Resting	Activated	Resting	Activated	Resting	Activated	Resting	Activated	Resting	Activated
Young	%	30.9	25.0	30.6	21.3	26.6	35.1	11.9	18.6	53.6	46.4
	#	502	351	496	298	431	492	193	261	1622	1402
	Range	(0–438)	(0–328)	(0–420)	(0–266)	(0–359)	(0–453)	(0–184)	(0–250)	(0–1401)	(0–1297)
Old	%	12.5	13.3	5.6	16.7	48.7	45.0	33.2	25.0	86.7	13.3
	#	49	8	22	10	191	27	130	15	392	60
	Range	(0–43)	(0–8)	(0–13)	(0–7)	(0–191)	(0–27)	(0–130)	(0–15)	(0–377)	(0–50)
Overall	%	27.4	24.6	25.7	21.1	30.9	35.5	16.0	18.9	57.9	42.1
	#	551	359	518	308	622	519	323	276	2014	1462
Mean % per mare ^‡^		5.4 ± 3.4	4.8 ± 3.1	4.5 ± 3.1	4.7 ± 2.5	9.4 ± 6.2	10.0 ± 6.4	5.6 ± 4.2	5.5 ± 3.5	7.2 ± 4.9	5.3 ± 4.6
Mean # per mare ^‡^		68.9 ± 53.3	44.9 ± 40.5	64.8 ± 51.1	38.5 ± 32.6	77.8 ± 46.7	64.9 ± 55.7	40.4 ± 26.0	34.5 ± 30.8	251.8 ± 170.5	182.8 ± 159.5

^‡^ No differences (*P* > 0.05) were observed between ovarian locations within the same developmental stages, between developmental stages within the same ovarian locations, or when ovarian locations were pooled (overall) between developmental stages. A total of 206 histological sections were evaluated (25.8 ± 1.1 sections per mare). Descriptive percentages were calculated within a row for the same age group and developmental stage. All mean data were analyzed using one-way ANOVA after arc sin square root transformation for percentage data or log_10_ transformation for non-percentage data.

**Fig 3:**
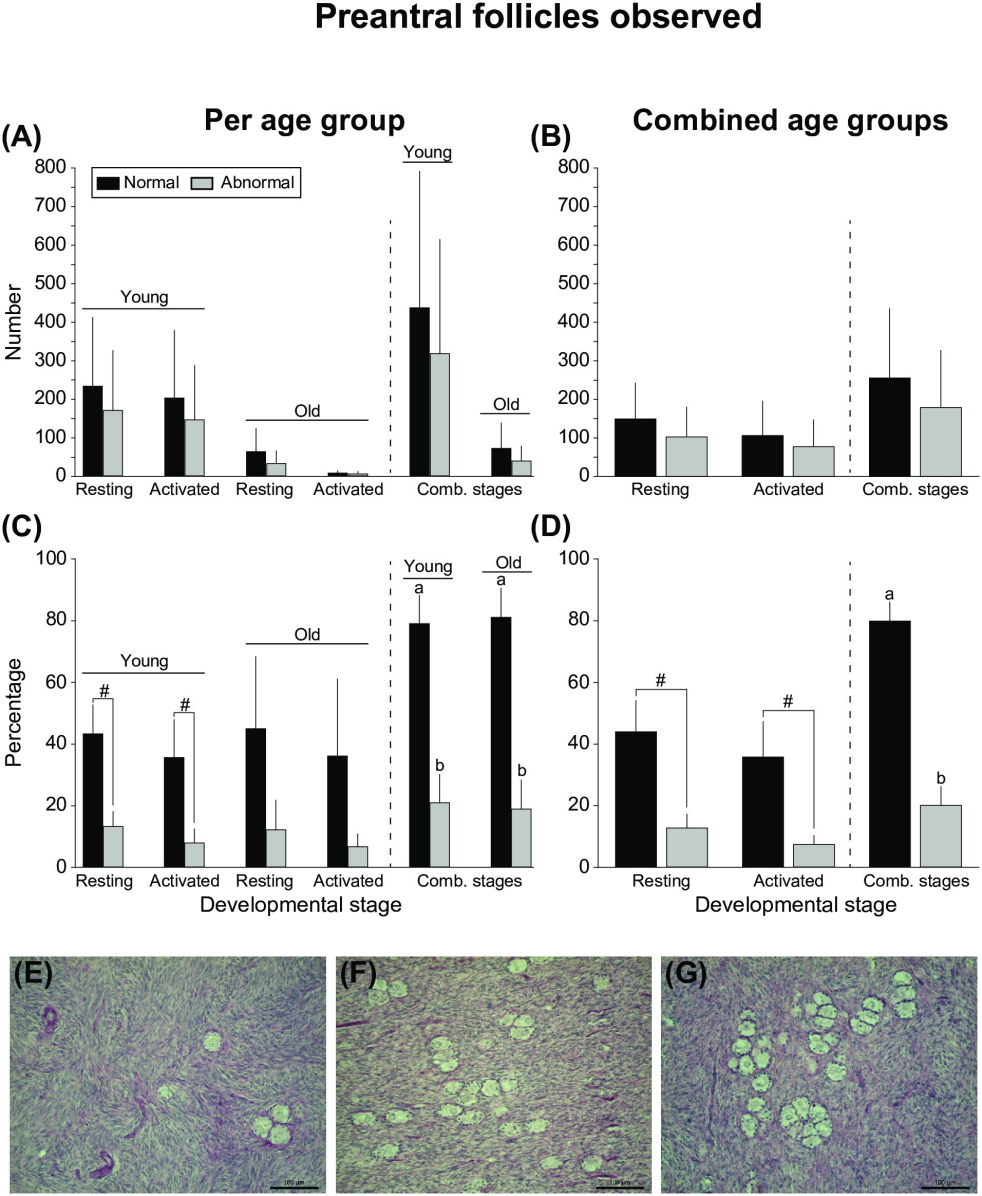
(A, B) Mean numbers (± SEM) and (C, D) mean percentages (± SEM) of observed resting and activated preantral follicles according to morphology considering (A, C) mare age and (B, D) when ages were combined. ^a,b^ Within the same age group or when ages were combined, and within the same developmental stage, values without a common superscript differed. ^#^ Indicates tendency (*P* = 0.05–0.09) within the same developmental stage. No differences (*P* > 0.05) were found regarding the number of observed follicles for any of the end points evaluated. Additionally, no differences (*P* > 0.05) in percentages were observed within the young and old age groups between developmental stages or between age groups within the same morphology and developmental stage. All data were analyzed using one-way ANOVA after log_10_ transformation for non-percentage data or arc sin square root transformation for percentage data. Comb., combined. (E–G) Representative images of randomly generated fields depicting (E) low numbers of follicles with few neighbors, (F) high numbers of follicles with a moderate number of neighbors, and (G) high numbers of follicles with a high number of neighbors are shown. Scale bars = 100 μm, magnification = 20×.

The numbers and percentages of observed resting and activated preantral follicles (Fig 3) according to follicular morphology per age group (Fig 3A and 3C) and when ages were combined (Fig 3B and 3D) are presented. Both young and old mares had higher (*P* < 0.05) percentages of morphologically normal follicles than abnormal follicles when developmental stages were combined (Fig 3C). Furthermore, when follicles were pooled for both mare age and developmental stage, the percentage of morphologically normal follicles was higher (*P* < 0.001) than that of abnormal follicles (Fig 3D).

### Neighborhood pattern identification and assessment

#### Number of follicles with neighbors

Considering only follicles with neighbors, a total of 2,931 preantral follicles were recorded. From these follicles, 1,656 primordial, 1,275 transitional, and no primary follicles were identified. Of the preantral follicles with neighbors, the numbers and percentages according to mare ages, ovarian locations (portions and regions), and follicular developmental stages are shown (Table 2). A wide range and large number of preantral follicles with neighbors were observed in the young mares (e.g., 0–410 and 2,626), while a narrow range and small number were observed in the old mares (e.g., 0–137 and 305). Out of the total number of follicles with neighbors, 89.6% came from young mares and 10.4% came from old mares. The numbers and percentages of follicles with neighbors were not affected (*P* > 0.05) by region, portion, or mare age (S1 Table).

**Table 2:** Descriptive percentages and numbers of follicles with neighbors according to mare ages, ovarian locations (portions and regions), and follicular developmental stages, and mean percentages (± SEM) and numbers (± SEM) of follicles with neighbors according to ovarian locations and follicular developmental stages.

Age group		Ovarian location (portion and region)									
		Lateral dorsal		Lateral ventral		Intermediary dorsal		Intermediary ventral		Overall	
		Resting	Activated	Resting	Activated	Resting	Activated	Resting	Activated	Resting	Activated
Young	%	30.9	23.8	30.1	21.3	26.2	35.1	12.8	19.8	52.9	47.1
	#	429	295	418	264	363	434	178	245	1388	1238
	Range	(0–382)	(0–273)	(0–365)	(0–241)	(0–322)	(0–410)	(0–173)	(0–237)	(0–1242)	(0–1161)
Old	%	9.0	16.2	3.0	18.9	51.1	45.9	36.9	18.9	87.9	12.1
	#	24	6	8	7	137	17	99	7	268	37
	Range	(0–24)	(0–6)	(0–8)	(0–7)	(0–137)	(0–17)	(0–99)	(0–7)	(0–260)	(0–30)
Overall	%	27.4	23.6	25.7	21.3	30.2	35.4	16.7	19.8	56.5	43.5
	#	453	301	426	271	500	451	277	252	1656	1275
Mean % per mare ^‡^		5.0 ± 3.4	5.1 ± 3.1	4.1 ± 3.2	5.0 ± 3.1	9.7 ± 6.6	10.1 ± 6.5	6.2 ± 4.6	5.2 ± 3.0	7.1 ± 5.2	5.4 ± 4.9
Mean # per mare ^‡^		56.6 ± 46.8	37.6 ± 33.7	53.3 ± 44.7	33.9 ± 29.7	62.5 ± 40.7	56.4 ± 50.6	34.6 ± 23.2	31.5 ± 29.4	207.0 ± 151.4	159.4 ± 143.3

^‡^ No differences (*P* > 0.05) were observed between ovarian locations within the same developmental stages, between developmental stages within the same ovarian locations, or when ovarian locations were pooled (overall) between developmental stages. A total of 206 histological sections were evaluated (25.8 ± 1.1 sections per mare). Descriptive percentages were calculated within a row for the same age group and developmental stage. All mean data were analyzed using one-way ANOVA after arc sin square root transformation for percentage data or log_10_ transformation for non-percentage data.

#### Percentage of follicles with neighbors

**Fig 4:**
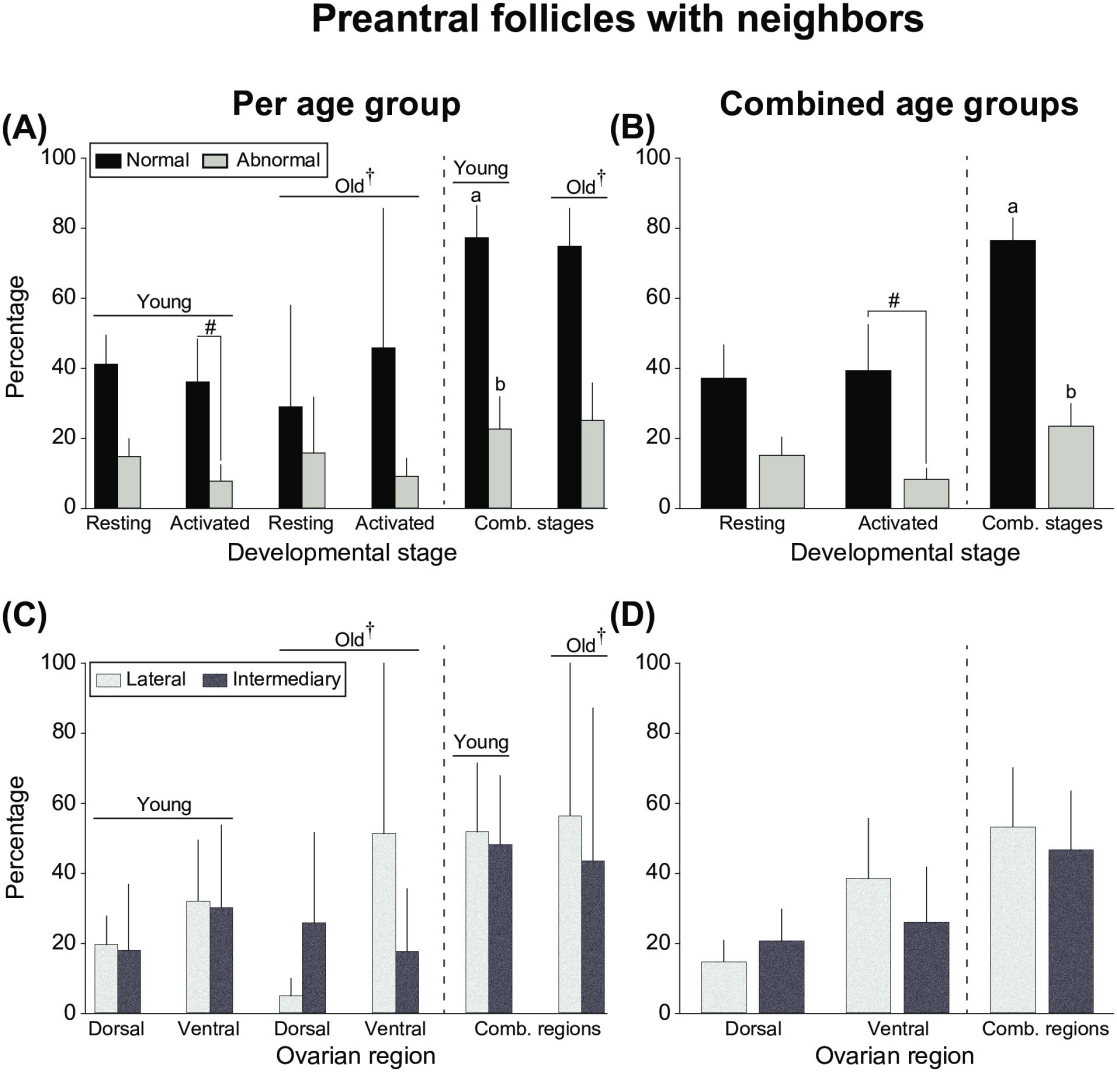
Mean percentages (± SEM) of preantral follicles with neighbors considering (A, B) morphology and developmental stage and (C, D) ovarian portions and regions (A, C) per age group and (B, D) with age groups combined. ^a,b^ Within combined stages, values without a common superscript differed. ^#^ Indicates tendency (*P* = 0.07–0.08) between morphologies within the activated developmental stage. ^†^ Statistical analyses could not be run on the old age group due to the low number (n ≤ 2) of old mares that had follicles with neighbors. No differences (*P* > 0.05) were observed for any of the end points considering resting follicles or ovarian portions and regions. All data were analyzed using one-way ANOVA after arc sin square root transformation. Comb., combined.

The mean percentages of follicles with neighbors (Fig 4) considering follicular morphology and developmental stage (Fig 4A and 4B) as well as ovarian portions and regions (Fig 4C and 4D) according to mare age (Fig 4A and 4C) and when ages were combined (Fig 4B and 4D) are shown. In young mares and in combined ages when follicles were pooled regarding developmental stage, a higher (*P* < 0.05) percentage of follicles with neighbors were normal than abnormal (Fig 4A and 4B). Specifically, when ages were combined (Fig 4B), 76.5 ± 6.5% of follicles were normal, while 23.5 ± 6.5% were abnormal. Interestingly, although there was a tendency (*P* < 0.07–0.08) for higher percentages of activated follicles to be normal versus abnormal in young mares and in combined ages, no differences (*P* > 0.05) were observed regarding resting follicles. Furthermore, percentages of follicles with neighbors did not differ (*P* > 0.05) according to ovarian location (portion and region; Fig 4C and 4D).

#### Number of neighbors per follicle

**Fig 5:**
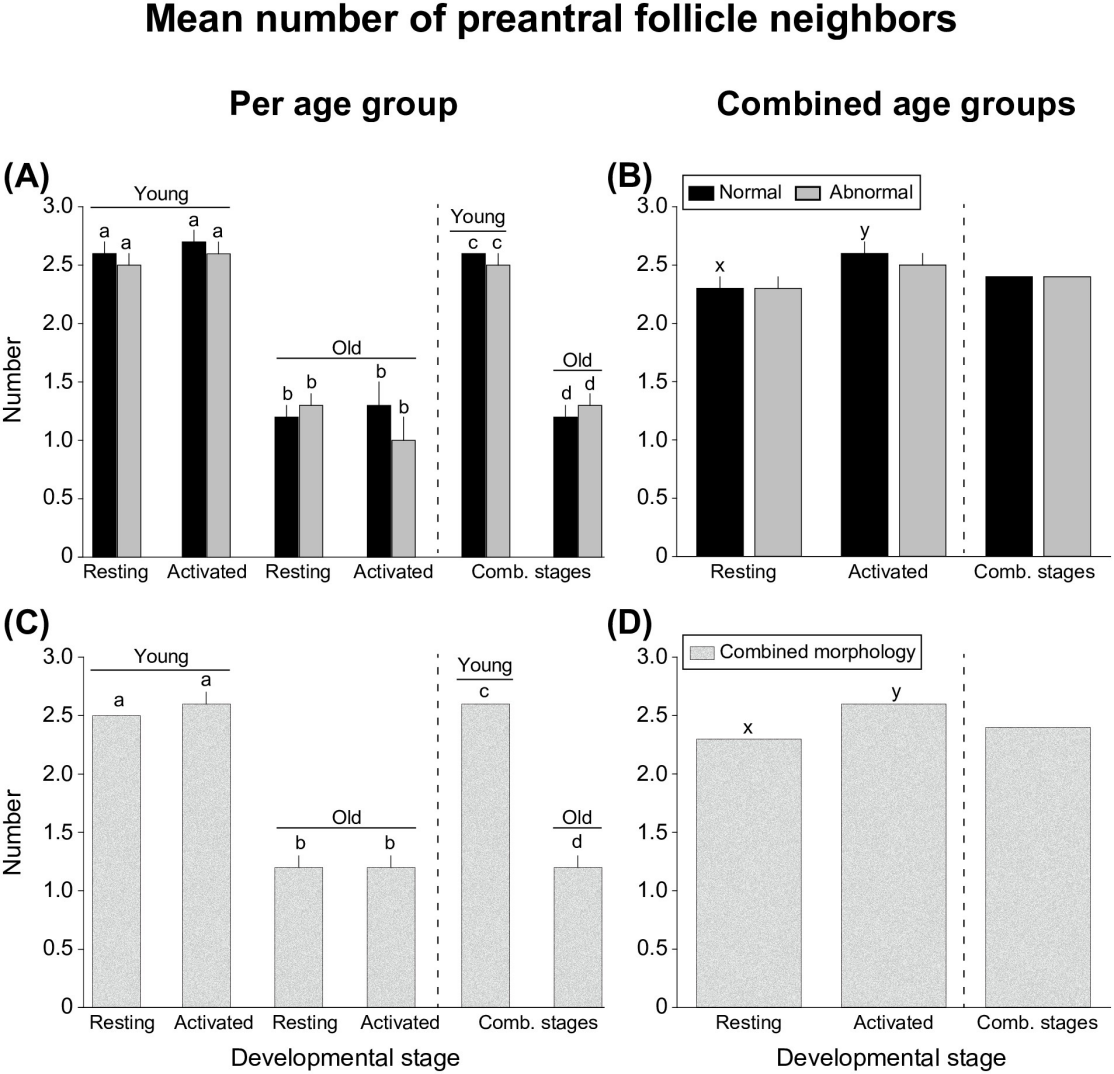
Mean number (± SEM) of neighbors per preantral follicle according to developmental stage for (A, B) morphology and for (C, D) combined morphologies (A, C) per age group and (B, D) when ages were combined. ^a,b^ Within the same developmental stage and morphology, values without a common superscript differed. ^c,d^ Within the same morphology when developmental stages were combined, values without a common superscript differed. ^x,y^ Between developmental stages and when ages were combined, values without a common superscript differed. No differences (*P* > 0.05) were observed when ages were combined comparing abnormal resting and activated follicles or between morphologies when developmental stages were combined. All data were analyzed using the Kruskal-Wallis test followed by Dunn’s test. Comb., combined.

The mean numbers of neighbors for a given preantral follicle considering morphology and developmental stage (Fig 5) according to mare age and when ages were combined are shown. The number of neighbors per follicle was greater (*P* < 0.001) in young mares than in old mares for all end points evaluated (Fig 5A and 5C). Specifically, when pooled for morphology and developmental stage (Fig 5C), the number of follicles per neighbor in young mares (2.6 ± 0.0) was greater (*P* < 0.001) than in old mares (1.2 ± 0.1). Overall (combined ages; Fig 5B), normal activated preantral follicles (2.6 ± 0.1) had greater (*P* < 0.001) numbers of neighbors than normal resting follicles (2.3 ± 0.1); however, the number of neighbors per follicle did not differ (*P* > 0.05) for abnormal follicles (Fig 5B). When pooled for both mare age and morphology, activated follicles had greater (*P* < 0.001) numbers of neighbors per follicle than resting follicles (Fig 5D).

**Fig 6:**
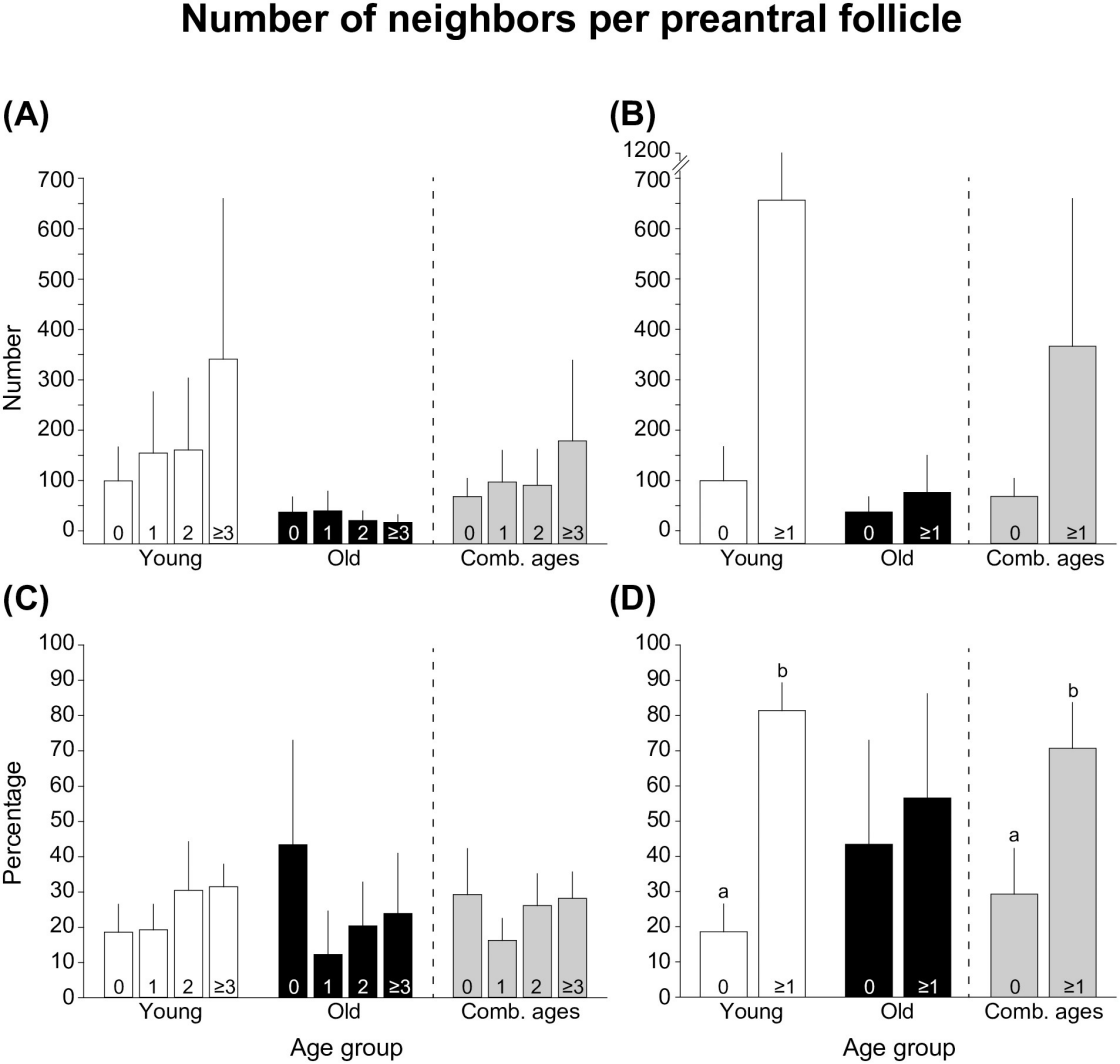
(A, B) Mean numbers (± SEM) and (C, D) mean percentages (± SEM) of preantral follicles with (A, C) 0, 1, 2 or ≥3 and (B, D) 0 or ≥1 neighbors according to age group and in combined ages. ^a,b^ Within the same age group, values without a common superscript differed. No differences (*P* > 0.05) were found comparing any of the end points evaluated considering numbers of follicles, percentages of follicles with 0, 1, 2, or ≥3 neighbors, or within the old age group. All data were analyzed after log_10_ transformation for non-percentage data or arc sin square root transformation for percentage data using one-way ANOVA followed by Fisher’s Least Significant Difference test. Comb., combined.

The mean numbers and mean percentages of preantral follicles with 0, 1, 2, ≥3, or grouped ≥1 neighbors according to mare age are shown (Fig 6). In young mares and combined ages, a higher (*P* < 0.05) percentage of follicles with ≥1 neighbor versus 0 neighbors was observed (Fig 6D). However, within the old age group, the percentages of follicles with ≥1 neighbor and 0 neighbors did not differ (*P* > 0.05). When follicles were analyzed for each developmental stage (S1 Fig), young mares had higher (*P* < 0.05) percentages of follicles with ≥1 neighbor than 0 neighbors, while no differences (*P* > 0.05) were observed in combined ages.

#### Distances between follicle neighbors

**Fig 7:**
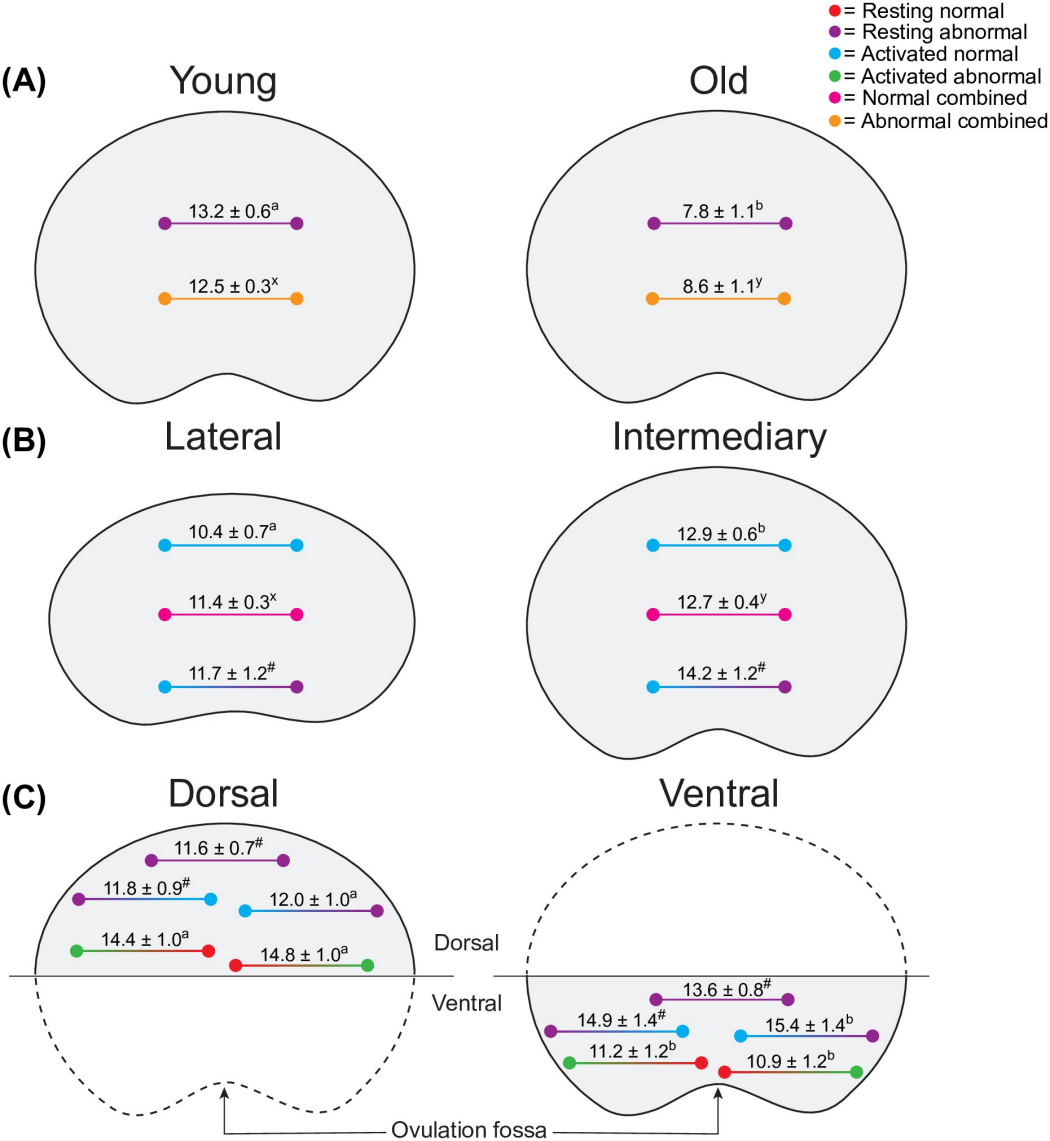
Illustration of the observed significant differences in mean distance (± SEM) between pairs of neighboring follicles of particular morphology and developmental stage considering (A) mare age groups, (B) ovarian portions, and (C) ovarian regions. ^a,b^ Within the same follicular morphology and developmental stage between pairs of neighboring follicles, values without a common superscript differed. ^x,y^ Within the same follicular morphology when developmental stages were combined between pairs of neighboring follicles, values without a common superscript differed. ^#^ Indicates tendency (*P* = 0.06–0.07) within the same follicular morphology and developmental stage between pairs of neighboring follicles. For the shown data, no differences (*P* > 0.05) were found within each age group, ovarian portion, or ovarian region. All data were analyzed using the Kruskal-Wallis test followed by the Dunn’s test. Comb., combined.

The different (*P* < 0.05) mean distances between preantral follicle neighbors according to morphology and developmental stage when compared between mare ages, ovarian portions, and ovarian regions are shown (Fig 7). Regarding the distances between neighboring follicles in young and old mares (Fig 7A), anchoring abnormal resting follicles were farther (*P* < 0.01) from abnormal resting neighbors in young mares. Additionally, when follicles were pooled considering developmental stage, neighboring abnormal follicles were farther (*P* < 0.05) from each other in young mares when compared to old mares.

Moreover, a greater (*P* < 0.01) distance between neighboring activated normal follicles was observed in the intermediary portion compared to the lateral portion (Fig 7B). Furthermore, when follicles were pooled for developmental stage, a greater (*P* < 0.01) distance was observed between neighboring normal follicles in the intermediary portion compared to the lateral portion. Additionally, the mean distances between anchoring activated normal follicles and resting abnormal neighbors in the intermediary portion tended (*P* = 0.08) to be greater than in the lateral portion.

Between the dorsal and ventral regions (Fig 7C), the distances between anchoring activated abnormal follicles and resting normal neighbors, as well as between anchoring resting normal follicles and activated abnormal neighbors, were both greater (*P* < 0.05) in the dorsal region. Furthermore, the distances between anchoring activated normal follicles with resting abnormal neighbors were greater (*P* < 0.05) in the ventral region than in the dorsal region. Additionally, distances between anchoring resting abnormal follicles with resting abnormal neighbors, as well as between anchoring resting abnormal follicles with activated normal neighbors, tended to be greater (*P* = 0.06–0.07) in the ventral region compared to the dorsal region. No differences (*P* > 0.05) were observed within each age group (Fig 7A), ovarian portion (Fig 7B), or ovarian region (Fig 7C) for mean distance to neighboring follicles when morphology and developmental stage were considered.

#### Relative probabilities of activation and odds ratios

Association analyses (relative probabilities of activation and odds ratios) between developmental stage and presence of follicle neighbors are shown (Table 3). The relative probability of activation (RPA), which compares the likelihood of an activated follicle to lack or have neighbors, tended (*P* = 0.09) to be higher in old mares (1.3 times more likely to lack neighbors) than in young mares (0.9 times more likely). However, the RPAs considering all individual and paired analyses (i.e., between morphologies, portions, or regions) performed were non-significant (*P* > 0.05). Overall, considering the odds of lacking neighbors between resting and activated developmental stages (OR), an activated follicle had 0.7 times lower (*P* < 0.0001) odds of lacking neighbors than a resting follicle. Within the young age group, an activated follicle had lower (*P* < 0.05) odds of lacking neighbors than a resting follicle; however, no differences (*P* > 0.05) were observed in the old age group. Additionally, activated follicles of young mares tended (*P* < 0.1) to have lower odds of lacking neighbors than those of old mares. A morphologically normal activated follicle had lower (*P* < 0.0001) odds of lacking neighbors than a morphologically normal resting follicle, while no differences (*P* > 0.05) were observed for morphologically abnormal follicles. Additionally, the odds for activated follicles to lack neighbors compared to resting follicles were lower (*P* < 0.05) or tended (*P* = 0.06) to be lower in the intermediary and lateral portions, respectively. Regarding the dorsal and ventral regions, activated follicles also had lower (*P* < 0.05) odds of lacking neighbors than resting follicles in both regions.

**Table 3:** Association analyses between developmental stages and presence of follicle neighbors.

	Activated	Resting	RPA (95% CI)	RPA *P*-value	OR (95% CI)	OR *P*-value
***Overall***			0.8 (0.0–12.3)	NS	0.7 (0.6–0.8)	***
Without neighbors	188	357				
With neighbors	1275	1656				
***Young mares***			0.9 (0.1–11.8)^†^	NS	0.8 (0.6–1.0)^†^	*
Without neighbors	165	233				
With neighbors	1238	1388				
***Old mares***			1.3 (0.0–67.1)	NS	1.3 (0.8–2.4)	NS
Without neighbors	23	124				
With neighbors	37	268				
***Normal follicles***			0.7 (0.0–11.9)	NS	0.6 (0.5–0.8)	***
Without neighbors	110	235				
With neighbors	734	956				
***Abnormal follicles***			0.9 (0.1–13.1)	NS	0.8 (0.6–1.1)	NS
Without neighbors	78	122				
With neighbors	541	700				
***Intermediary portion***			0.7 (0.0–10.8)	NS	0.6 (0.5–0.8)	**
Without neighbors	93	168				
With neighbors	705	777				
***Lateral portion***			0.9 (0.0–14.4)	NS	0.8 (0.6–1.0)	^#^
Without neighbors	95	189				
With neighbors	570	879				
***Dorsal region***			0.8 (0.1–12.5)	NS	0.7 (0.6–0.9)	*
Without neighbors	127	219				
With neighbors	750	955				
***Ventral region***			0.7 (0.0–12.0)	NS	0.6 (0.4–0.8)	**
Without neighbors	61	138				
With neighbors	525	701				

RPA, relative probability of activation (number activated follicles / number activated follicles + number resting follicles); OR, odds ratio; CI, confidence interval; NS, non-significant.

* *P* < 0.05;

** *P* < 0.01;

*** *P* < 0.001.

^#^ *P* = 0.06.

^**†**^ RPA (*P* = 0.09) and OR (*P* = 0.09) between age groups (young *vs*. old). No differences (*P* > 0.05) were observed between the RPAs or ORs for any other group (normal *vs*. abnormal, intermediary *vs*. lateral, dorsal *vs*. ventral).

### Evaluation of preantral follicle clustering

The Morisita indices of clustering (I_δ_) for preantral follicles according to ovarian regions and portions in each age group are shown (Table 4). Significant clustering patterns (Z > 1.96) were indicated in all areas of the ovary, regardless of mare age, ovarian portion, or ovarian region (I_δ_ range = 5.1–7.7). Young mares showed more (*P* < 0.05) follicular clustering than old mares in the dorsal region, within the lateral portion, but not in the intermediary portion (*P* > 0.05). Furthermore, higher (*P* < 0.05) follicular clustering was observed in the young mares overall.

**Table 4:** Preantral follicle clustering index according to ovarian regions and portions in mares of different ages.

Ovarian region	Age group					
	Young		Old		Combined ages	
	Lateral	Intermediary	Lateral	Intermediary	Lateral	Intermediary
Dorsal	7.1 ± 1.6^a^	5.5 ± 0.9	6.2 ± 3.6^b^	7.2 ± 2.6	6.8 ± 1.6	6.2 ± 1.1
***Dorsal combined***	6.4 ± 1.0		6.7 ± 2.2		6.5 ± 1.0^#^	
Ventral	7.7 ± 1.5	7.4 ± 1.0	6.7 ± 2.2	5.1 ± 1.2	7.5 ± 1.3	6.6 ± 0.8
***Ventral combined***	7.6 ± 1.0		5.8 ± 1.2		7.1 ± 0.8	
Combined regions	7.5 ± 1.1	6.5 ± 0.7	6.4 ± 2.2	6.2 ± 1.5	7.2 ± 1.0	6.4 ± 0.7
***Combined regions and portions***	7.1 ± 0.7^A^		6.3 ± 1.3^B^		6.8 ± 0.6	

Preantral follicle clustering was evaluated using Morisita’s index of clustering (I_δ_) with all indices testing positive for clustering (Z ≥ 1.96).

^a,b^ Within the same ovarian region and portion, values without a common superscript differed (*P* < 0.05).

^A,B^ Within combined ovarian regions and portions, values without a common superscript differed (*P* < 0.05).

^#^ The dorsal ovarian region tended to differ (*P* = 0.06) from the ventral ovarian region when ages and ovarian portions were combined. No differences (*P* > 0.05) were observed between age groups within the intermediary portion and dorsal region of the ovary, within the ventral region and combined regions, or between portions when ages were combined. Data were analyzed using the Kruskal-Wallis test.

The ventral region tended (*P* = 0.06) to have higher clustering than the dorsal region when ages were combined; however, no differences (*P* > 0.05) were observed between and within ovarian portions.

## Discussion

The present study documented, for the first time in any livestock species, crowding patterns of preantral follicles in the ovarian cortex. Furthermore, these crowds were also characterized according to follicular morphology and location in portions and regions of the ovary for the first time in any species. The main findings of the present study demonstrated that follicular clustering is observed in the mare ovary, similar to that of women and mice, and that the degree of clustering depends upon mare age and ovarian location. Additionally, it was shown that follicles with neighbors (other follicles within a 40 μm radius) were overall more likely to be morphologically normal. Furthermore, young mares were observed to have a higher mean number of neighbors per follicle than old mares. Finally, it was found that morphologically normal activated follicles had a higher number of neighbors and increased odds of having neighbors than normal resting follicles. Altogether, these results provide valuable insight into the dynamic nature of early preantral follicles in the mare ovary, as well as vital information to be used for ARTs involving early folliculogenesis.

Considering clustering of preantral follicles using Morisita’s index of clustering, all areas of the mare ovary indicated clustering, regardless of mare age, ovarian portion, or ovarian region. Clustering of preantral follicles in the ovary has been documented in both mice [20, 29, 30] and women [20, 31]. Interestingly, the aforementioned studies reported that follicular clustering increases with age in both species, perhaps due to expected decreases in follicle density and regular follicular activity over time [31]. However, in the present study, it was found that clustering decreased with age, a finding that is supported by the fact that in old mares, the spatial distribution of follicles exhibits a more dispersive pattern [18, 19]. The working hypothesis of ovarian plasticity, in which preantral follicles are suggested to migrate throughout the ovarian cortex, postulates that the rigidity of ovarian tissue is a key component of this migration [17]. It has been proposed that primordial follicles may migrate from the more rigid outer regions of the ovarian cortex to the softer, less rigid regions of the cortex, closer to the ovarian geometric center, as they initiate and continue development to the primary stage [17–19, 30]. However, it has been shown in the ovaries of women that levels of substances that contribute to rigidity and elasticity of the ovarian extracellular matrix (i.e., collagen, elastin, fibrillin) change as aging progresses [32–34]. Thus, we hypothesize that the age-related decrease in follicular clustering in the mare is perhaps due to age-related changes in the rigidity of the ovarian tissue. Furthermore, the different clustering patterns observed in this study may be associated with the inversion of the ovarian cortical layer observed in the mare. Specifically, a decrease in clustering in older mares was observed in the lateral portion and dorsal region of the ovary. Previous work by our laboratory showed that the lateral portion and dorsal region, an outer location of the ovary proposed to be more rigid [17, 30], had a high density of preantral follicles in young mares [19]. It is known that overall collagen levels in the ovary increase and levels of hyaluronic acid, elastin, and fibrillin decrease with age in mice and women [33, 34], leading to increased ovarian tissue rigidity; however, it is unknown if this rigidity is homogenous. Perhaps over time in the mare, age-related increases in ovarian rigidity are heterogenous, with some areas becoming stiffer and the lateral and dorsal location specifically experiencing significant decreases in rigidity, leading to decreased follicular clustering in this area.

Additionally, in the present study, preantral follicles tended to cluster more in the ventral region overall. This finding may be related to the presence of the ovulation fossa in the ventral region, a place that large antral/preovulatory follicles must continually migrate toward for ovulation to occur in the mare. Perhaps during growth and migration, the preovulatory follicles may push clustered preantral follicles in the ventral region closer together. Meanwhile, the clusters of preantral follicles not deeply located within the ventral region are pushed into the dorsal region, leading to increased follicular density in the dorsal region [19]. Further studies that assess clustering and tissue rigidity throughout different times of the estrous cycle, proximity to the ovulation fossa, and in pre-pubertal fillies are appealing to confirm the relationship between tissue rigidity and preantral follicle clustering.

Another novel finding of the current study was that the presence of follicle neighbors and morphology seem to be linked. Out of all the follicles observed in the study, 82% of primordial follicles and 87% of transitional follicles had neighbors, with a large majority of these follicles with neighbors being morphologically normal. Several paracrine factors known to be associated with follicular activation, including bone morphogenic proteins, PI3K, PDK1, and mTOR, have also been linked to survivability of primordial follicles [7, 8]. Thus, we hypothesize that the presence of neighbors is important not only for proper activation of preantral follicles to grow, but also for maintenance of normal morphology and follicular survivability. Furthermore, when assessing the distribution of preantral follicles with neighbors across ovarian regions and portions, no differences were observed. These findings suggest that the tendency for increased clustering in the ventral region is due not to paracrine factors but likely to spatial effects of large antral/preovulatory follicles, as previously hypothesized. Moreover, the low number of old mares that had preantral follicles with neighbors made analyses of data from this group alone difficult; therefore, future studies focused on assessing follicle neighborhood patterns in greater numbers of old mares are encouraged.

It was observed that follicles from young mares had more neighbors on average than those of old mares, regardless of developmental stage or follicular morphology. This result was expected, as a greater number of follicles in young females is a shared characteristic among several species (women: [35, 36]; bovine: [37]; equine: [19]; macaques: [38]; deer: [14]; mice: [39]). Additionally, when age groups were combined, morphologically normal activated follicles had greater numbers of neighbors than normal resting follicles. It has been suggested that clusters of primordial follicles are activated to develop and migrate together [30], and if any of these developing follicles become morphologically abnormal, migration stops [19]. Thus, we hypothesize that follicles that are activated to develop migrate together, closer to the ovarian geometric center, leading to the increased mean number of neighbors observed in activated follicles. As these activated follicles converge toward the geometric center, resting follicles are left with fewer mean neighbors remaining in the more lateral areas of the ovarian cortex. Furthermore, any follicles that become morphologically abnormal will stop migrating, leaving these follicles more dispersed through the ovarian cortex. Additionally, the higher odds of a morphologically normal activated follicle to have neighbors supports our finding that normal activated follicles have more neighbors than normal resting follicles. Interestingly, it has been suggested that, in mice and women, the number of neighbors decreases as follicles develop after the early primary stage [20]. Future *in vitro* studies that assess morphology of activated follicles in the presence or absence of neighbors, as well as studies that use ovarian biopsy techniques to obtain tissue samples at different distances from the geometric center, are warranted to continue to test our hypothesis.

To the best of our knowledge, no study to date has assessed preantral follicle clustering as it relates to ovarian portions/regions and age or evaluated follicular neighborhood patterns according to age, follicular morphology and developmental stage, and ovarian portions/regions. Thus, by combining the hypotheses generated using results from the current study, we propose the following working hypothesis to postulate the mechanisms behind our results. The mare ovarian cortex may follow a gradient of rigidity, with the outer regions of the cortex being more rigid than the less rigid ovarian geometric center. This rigidity, along with paracrine factors that promote dormancy, keep resting (primordial) follicle clusters in the outer regions of the ovarian cortex. Some of these resting follicle clusters will be synchronously activated to develop and begin migrating down the rigidity gradient to the geometric center while simultaneously prioritizing production of morphology maintenance/atresia resistance factors over dormancy factors. Nevertheless, several follicles in each migrating cluster will become morphologically abnormal and be left behind as the normal follicles continue to develop and migrate to the geometric center. As aging occurs in the mare, rigidity will decrease markedly in the lateral and dorsal area of the ovary and promote a more dispersed pattern of preantral follicles in this area in particular, while tissue rigidity around the geometric center will increase, leading to a more uniform tissue rigidity gradient. Additionally, the cyclic growth and migration of large antral/preovulatory follicles in the ventral region promotes a slightly higher pattern of clustering in this region, with clusters deeper being pushed further together while more outer clusters are pushed into the dorsal region.

In conclusion, the present study reports that follicular clustering does indeed occur in the mare and that this clustering changes with age and ovarian location. Furthermore, a relationship between normal follicle morphology and the presence of neighbors was discovered, along with a decreased number of follicle neighbors as a mare ages. Additionally, it was found that morphologically normal activated follicles were more likely to have neighbors, and that these follicles had higher numbers of neighbors than normal resting follicles. Altogether, our results provide further support for ovarian plasticity and age-related changes within the equine ovary, and our working hypothesis attempts to explain some of the mechanisms behind our results. These findings have many potential applications not only for ARTs (i.e., targeted ovarian biopsies, follicle culture, ovarian tissue supplementation) but also for comparative analysis of mare ovarian tissue with other species. The findings from the current study could be used to collect follicle-dense ovarian tissue, such as from the lateral and dorsal area of the young mare ovary, and perhaps the follicles from this tissue can be cultured to explore if specific conditions (i.e., number of follicle neighbors, decreasing media rigidity, etc.) may allow for targeted activation. If the conditions for maintaining normal, healthy primordial follicles in dormancy or for controlled activation into primary and later follicular stages are met, then the majority population of preantral follicles could be better utilized for ARTs and infertility treatments. Future studies that assess rigidity of equine ovarian tissue and follicular clustering during the estrous cycle and in pre-pubertal fillies and a high number of old mares are warranted. Furthermore, in-depth molecular studies assessing differences in paracrine factors produced by resting and early activated follicle clusters are encouraged to elucidate mechanisms that control preantral folliculogenesis in the mare.

## Supporting information

S1 Fig Mean percentages (± SEM) of resting and activated preantral follicles with 0 or ≥1 neighbors according to age group and in combined ages. ^a,b^ Within the same age group and developmental stage, values without a common superscript differed. ^†^ Statistical analyses could not be run on the resting follicles within the old age group due to the low number of animals (n ≤ 2) that had resting follicles. All data were analyzed after arc sin square root transformation using one-way ANOVA followed by Fisher’s Least Significant Difference test.
(PDF)

S1 Table Mean percentages (± SEM) and numbers (± SEM) of follicles with neighbors according to mare ages and ovarian locations (portions or regions). (PDF)

S1 File (XLSX)
