# Supplementary figures and images for: Characterization of preantral follicle clustering and neighborhood patterns in the equine ovary
Source: PLoS One. 2022 Oct 4;17(10):e0275396. doi: 10.1371/journal.pone.0275396 (PMC9531796; doi:10.1371/journal.pone.0275396)

Neighbors per preantral follicle

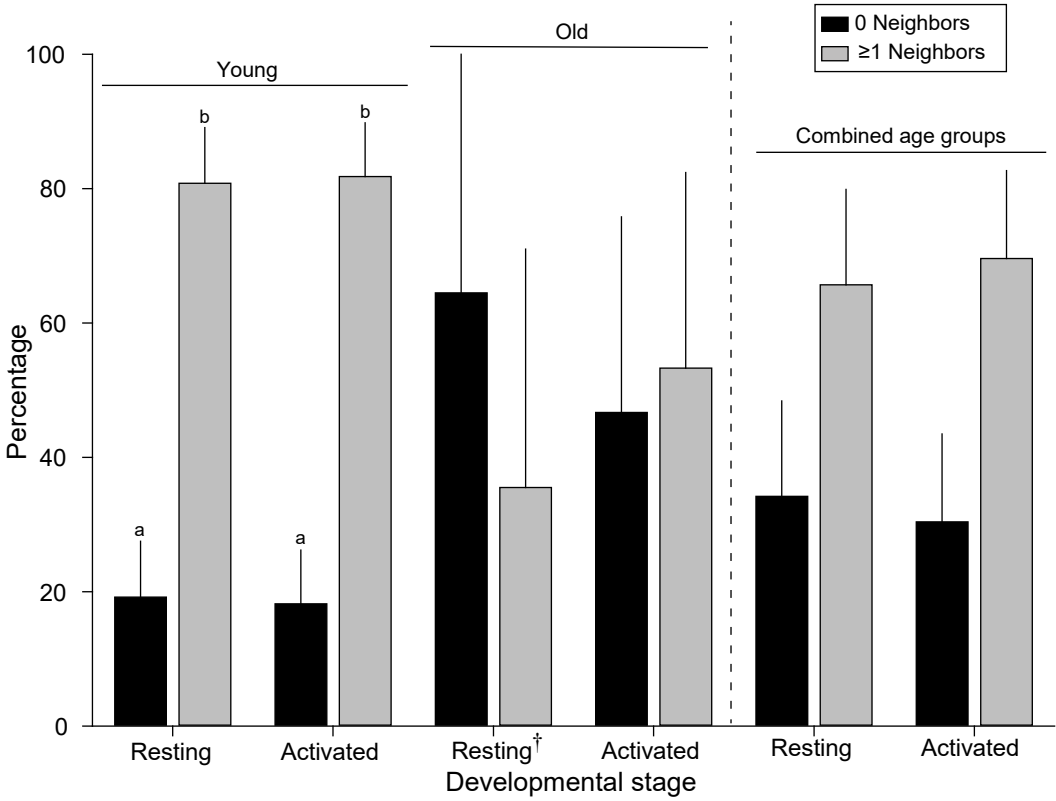

Figure S1

Supplement: S1 Fig — a,b Within the same age group and developmental stage, values without a common superscript differed. † Statistical analyses could not be run on the resting follicles within the old age group due to the low number of animals (n ≤ 2) that had resting follicles. All data were analyzed after arc sin square root transformation using one-way ANOVA followed by Fisher’s Least Significant Difference test. (PDF) [file pone.0275396.s001.pdf]
