# Supplementary material for: Characterization of preantral follicle clustering and neighborhood patterns in the equine ovary
Source: PLoS One. 2022 Oct 4;17(10):e0275396. doi: 10.1371/journal.pone.0275396 (PMC9531796; doi:10.1371/journal.pone.0275396)
Supplement: S1 Table — (PDF) [file pone.0275396.s002.pdf]

S1 Table. Mean percentages (± SEM) and numbers (± SEM) of follicles with neighbors according to mare ages and ovarian locations (portions or regions)

Developmental Stage		Mean (± SEM) follicles per mare					
		Age group		Ovarian portion		Ovarian region	
		Young	Old	Lateral	Intermediary	Dorsal	Ventral
Resting ‡	%	13.2 ± 11.4	22.0 ± 22.0	6.6 ± 5.5	5.9 ± 3.8	7.2 ± 5.2	5.3 ± 4.0
	#	347.0 ± 299.1	67.0 ± 67.0	109.9 ± 91.4	97.1 ± 63.7	119.1 ± 86.0	87.9 ± 65.6
Activated ‡	%	11.8 ± 10.8	3.0 ± 2.3	5.6 ± 5.0	6.9 ± 6.3	7.4 ± 6.6	5.1 ± 4.6
	#	309.5 ± 284.1	9.3 ± 7.1	71.5 ± 63.4	87.9 ± 80.0	94.0 ± 84.3	65.4 ± 59.0
Overall ‡	%	22.4 ± 19.9	2.6 ± 2.5	6.2 ± 5.3	6.3 ± 4.8	7.3 ± 5.8	5.2 ± 4.2
	#	656.5 ± 583.2	76.3 ± 73.9	181.4 ± 154.7	185.0 ± 140.3	213.1 ± 169.6	153.3 ± 124.0

‡ No differences ($P > 0.05$) were observed between age groups within the same developmental stages or overall, between ovarian portions within the same developmental stages or overall, or between ovarian regions within the same developmental stages or overall. Furthermore, no differences ($P > 0.05$) were observed between developmental stages within the same age group, ovarian portion, or ovarian region. All data were analyzed using one-way ANOVA after arc sin square root transformation for percentage data or $\log_{10}$ transformation for non-percentage data.
